# Outcomes of Vital Sign Monitoring of an Acute Surgical Cohort With Wearable Sensors and Digital Alerting Systems: A Pragmatically Designed Cohort Study and Propensity-Matched Analysis

**DOI:** 10.3389/fbioe.2022.895973

**Published:** 2022-06-27

**Authors:** Fahad Mujtaba Iqbal, Meera Joshi, Rosanna Fox, Tonia Koutsoukou, Arti Sharma, Mike Wright, Sadia Khan, Hutan Ashrafian, Ara Darzi

**Affiliations:** ^1^ Division of Surgery & Cancer, London, United Kingdom; ^2^ Department of Cardiology, West Middlesex University Hospital, Isleworth, United Kindom; ^3^ Innovation Business Partner, Chelsea and Westminster Hospitals NHS Foundation Trust, London, United Kingdom

**Keywords:** remote sensing technology, clinical trial, patient deterioration, patient deterioration detection, monitoring, ambulatory

## Abstract

**Background:** The implementation and efficacy of wearable sensors and alerting systems in acute secondary care have been poorly described.

**Objectives:** to pragmatically test one such system and its influence on clinical outcomes in an acute surgical cohort.

**Methods:** In this pragmatically designed, pre-post implementation trial, participants admitted to the acute surgical unit at our institution were recruited. In the pre-implementation phase (September 2017 to May 2019), the SensiumVitals™ monitoring system, which continuously measures temperature, heart, and respiratory rates, was used for monitoring alongside usual care (intermittent monitoring in accordance with the National Early Warning Score 2 [NEWS 2] protocol) without alerts being generated. In the post-implementation phase (May 2019 to March 2020), alerts were generated when pre-established thresholds for vital parameters were breached, requiring acknowledgement from healthcare staff on provided mobile devices. Hospital length of stay, intensive care use, and 28-days mortality were measured. Balanced cohorts were created with 1:1 ‘optimal’ propensity score logistic regression models.

**Results:** The 1:1 matching method matched the post-implementation group (n = 141) with the same number of subjects from the pre-implementation group (n = 141). The median age of the entire cohort was 52 (range: 18–95) years and the median duration of wearing the sensor was 1.3 (interquartile range: 0.7–2.0) days. The median alert acknowledgement time was 111 (range: 1–2,146) minutes. There were no significant differences in critical care admission (planned or unplanned), hospital length of stay, or mortality.

**Conclusion:** This study offered insight into the implementation of digital health technologies within our institution. Further work is required for optimisation of digital workflows, particularly given their more favourable acceptability in the post pandemic era. Clinical trials registration information: ClinicalTrials.gov Identifier: NCT04638738.

## Introduction

Prodromal alterations in vital parameters help identify clinical deterioration through early changes, often preceding adverse events (Goldhill et al., 1999; Buist et al., 2004; Cuthbertson et al., 2007; DeVita et al., 2010; Smith, 2010; Kenzaka et al., 2012; Hollis et al., 2016). Therefore, monitoring of these physiological parameters is considered a fundamental aspect of delivering clinical care. Despite this, clinical deterioration can go undetected with unfavourable consequences: late referrals to intensive care; worsened morbidity, and mortality (McQuillan et al., 1998; Culliane et al., 2005; Britain and N. P. S. A., 2007; Luettel et al., 2007; NICE, 2007; Findlay et al., 2012).

Vital signs, consisting of heart rate (HR), respiratory rate (RR), temperature, blood pressure (BP), oxygen saturations (& supplemental oxygen), and level of consciousness, are routinely but intermittently monitored for individuals admitted to non-intensive (general) hospital wards. The frequency of monitoring and subsequent action has been protocolised through the endorsement of the National Early Warning Score (NEWS 2) by The Royal College of Physicians. Each parameter is individually scored according to severity and combined for a total NEWS 2 score. Observations are performed every 4–6 h for those with a score of one to four, hourly for a score of five to seven, and continuously for patients with a NEWS 2 score of 7 or more. For acutely unwell individuals, an increased frequency of monitoring is required (Royal College of Physicians, 2017).

Early warning scores predicate that individuals at risk of deterioration are identified early through initial variations in vital parameters (e.g., elevated respiratory rate or reduced blood pressure), allowing for early intervention (Ridley, 2005). Indeed, their implementation has shown good predictive value on deterioration, positively influencing clinical outcomes (Downey et al., 2017). However, several limitations are recognised, critically their intermittent nature, allowing for acute deterioration in between measurements to be missed (Downey et al., 2017). Inadequate monitoring frequency and NEWS 2 calculations; transcribing errors from observation machines; misplacement of paper-based charts have, additionally, all been reported (Chen et al., 2009; Edwards et al., 2010; Christofidis et al., 2014).

Near real-time continuous monitoring of vital signs using novel wearable sensors, without hindering ambulation, may offer a solution to tackle these issues. A culmination of additional data points and reduced reliance on clinical personnel availability for performing observations may allow for earlier recognition of deterioration with reduced workload for healthcare staff (Tarassenko et al., 2006). Subsequent alerting mechanisms prompt healthcare professionals to act accordingly. These alerts are generated when pre-established thresholds for vital parameters, often configurable, are breached, informing healthcare professionals on mobile phone devices or central monitoring hubs. This, in time, can be used to predict deterioration rather than relying on protocol-driven intermittent traditional based measurements (Wells et al., 2022).

Although a meta-analysis supported the use of alerting systems in cases of sepsis to reduce hospital and intensive care length of stay (Joshi et al., 2019), another study reported there was a paucity in the literature with regards to the efficacy of wearable continuous monitoring on clinical outcomes (Leenen et al., 2020). Furthermore, an additional study reported no conclusive evidence to favour continuous monitoring outside of intensive care settings (Downey et al., 2018b). The heterogeneity of study designs, patient selection bias, complexity of interventions, and lack of controls, however, has limited meaningful conclusions. Moreover, the real-world applicability of such interventions is seldom reported, an essential component to understanding implementation of wearable solutions.

Recent experiences of nursing staff based on a surgical ward reported the potential utilitarian value of wearable sensors in providing individualised patient monitoring, aiding clinical decision making, and increasing efficiency in prioritising patients, particularly during busier shifts. However, they noted that not all patients may require continuous monitoring and a selection process should be in place that can be adapted to an individual’s needs (Areia et al., 2022).

To date, studies have focussed on demonstrating acceptability, feasibility, reliability, and experiences of wearable sensor based continuous monitoring (Hernandez-Silveira et al., 2015; Downey C. et al., 2018c; Breteler et al., 2019; Leenen et al., 2020; Weenk et al., 2020), with real-world incorporation of digital alerts into remote monitoring yet to be studied. As such, the aim of this work was to pragmatically test this in an acute surgical cohort, highlighting our implementation experience.

## Materials and Methods

### Study Design

This pragmatically designed, single-centre, pre-post implementation study was conducted on the acute surgical unit at our institution (West Middlesex University Hospital). This unit typically dealt with common surgical presentations referred from the emergency department or from primary care. These include s acute abdominal pain caused by a variety of pathologies (e.g., bowel obstruction, appendicitis, cholecystitis, diverticulitis) but also acute bleeding symptoms (e.g., haematuria, rectal bleeding) and was staffed by a rotating on-call system of healthcare professionals. Therefore, this represented a cohort with potential to rapidly deteriorate where the utilisation of digital alerts may be impactful. A medical cohort was considered but the rapid transfer of patients to other long stay wards risked a high rate of data attrition; given the financial and logistical needs to engineer the wards for digital alerting systems, as detailed in our published protocol, the surgical unit was favoured (Iqbal et al., 2021b). The pre-implementation phase dates from September 2017 to May 2019 and post-implementation phase from May 2019 to March 2020.

The pre-implementation phase involved using the SensiumVitals™ system in combination with usual care. However, healthcare staff were unable to view the sensor data and digital alerts were not generated. Usual care, in our institution, involved intermittent monitoring of vital signs in accordance with NEWS2.

In the post-implementation phase, alerting systems following recognition of abnormal parameters were activated. These alerts were transmitted to mobile devices and central monitoring hubs, with alert acknowledgement required from healthcare staff.

All participants provided informed consent. Ethical approval for this study was granted by the Yorkshire & The Humber—Leeds East Research Ethics Committee on 1st September 2017 (reference: 17/YH/0296; IRAS: 222979) and this trial was performed in accordance with Good Clinical Practice guidelines and the Declaration of Helsinki. Patient data were anonymised to ensure privacy. Storage and handling of personal data complied with the General Data Protection Regulation.

### Wearable Sensor and Alerting Thresholds

A disposable, lightweight, waterproof, wearable “patch” created by SensiumVitals™ was attached to a participant’s chest with two adhesive ECG electrodes, recording HR, RR and axillary temperature every 2 minutes. These data and any subsequent generated alerts were viewable and actionable through a secured web-browser or mobile device provided to healthcare staff. Patented embedded algorithms processed captured data to prevent reporting of noisy or irregular signals, thereby reducing false alerts. This consists of an initial stage of digital filtering, whereby unwanted artefacts are removed from recorded signals (i.e., from external sources or through muscular electrical activity); a decision-making stage follows this by ensuring the recorded signal meets a series of rules and empirically derived thresholds to allow the final value to be recorded and interpreted by the end user; this ensures a quality assurance check has been undertaken (Wong et al., 2009; Hernandez-Silveira et al., 2015). Further details have been described in our protocol (Iqbal et al., 2021b).

Alerts were generated when pre-established thresholds were breeched for 10 consecutive minutes for measured vital signs. A 10-min window was based on (unpublished) internal pilot testing data which tested periods ranging from 6 to 18 min. Ten minutes was chosen to balance the risk of alert fatigue to healthcare staff against the potential usefulness of alerts. These thresholds were individually tailorable but were initially programmed to trigger, in accordance with red (heart rate over 131 beats per minute, respiratory rate over 25 breaths per minute) and yellow (temperature of 38.1°C) NEWS2 cut-offs (Royal College of Physicians, 2017).

Subsequent actions taken by healthcare staff were recorded, these included: repeating a full set of observations; reviewing the clinical status of the participant; escalating for a review from a senior member of the healthcare team; re-adjusting the electrodes for improved data capture; initiating further treatment or following a protocol (e.g., sepsis 6 (Levy et al., 2010)); or taking no further action. The decision to act upon the alert remained at the clinical discretion of the healthcare professional who received the alert.

### Eligibility Criteria

Adults (aged over 18 years) admitted to the acute surgical unit and able to understand the participant information sheet were eligible for inclusion. Individuals with cardiac implantable electronic devices; a skin reaction to the wearable sensor or its components; an open chest wound; or those who withdrew consent were excluded from the study.

### Outcome Measures

Measurable outcomes included hospital length of stay, intensive care use (planned or unplanned), and 28-days mortality. Outcomes were obtained from case note review, SensiumVitals™ data, and electronic health records.

### Statistical Analysis

All values are expressed as median (range) or number (%). Categorical variables were compared using the chi-squared or Fisher’s exact test, dependent on the observations available. Non-parametric data were analysed using the Wilcoxon Rank Sum test. Odds ratio and incidence rate ratios were calculated for recorded outcome measures.

Propensity score matching (PSM) was used to estimate the effect of digital alerting on clinical outcomes accounting for confounding by the included covariates. Balanced cohorts were created using 1:1 “optimal” PSM logistic regression model (Austin, 2014). Included covariates were age, sex, ethnicity, American Society of Anaesthesiologists (ASA) grade, presenting NEWS 2 score, and comorbidities as per the Charlson Index (Armitage et al., 2010).

Balance diagnostics were conducted using standardised mean differences, with a value of ≤0.1 indicating good balance; love plots were generated to depict balance (Austin, 2014).

Data analysis was performed in RStudio version 3.6.3 (R Studio, Boston, MA, United States) with ggplot2, Matchit, gtsummary packages (Austin, 2014; Wickham et al., 2016; Sjoberg et al., 2021). A *p* value of <0.05 was deemed as statistically significant.

### Power Considerations

Formal power calculations on the primary outcome were not possible given the lack of data. A previous study estimated sample sizes of 325–625 to be appropriate; however, the results of the recruited 226 participants were not significant (Downey C. et al., 2018). We, therefore, aimed to recruit a minimum of 600 individuals.

Owing to the COVID-19 pandemic and restructuring of the acute surgical unit, this trial concluded prematurely, limiting our original planned sample size.

## Results

### Baseline Demographics and Balanced Cohort Assembly

**FIGURE 1:**
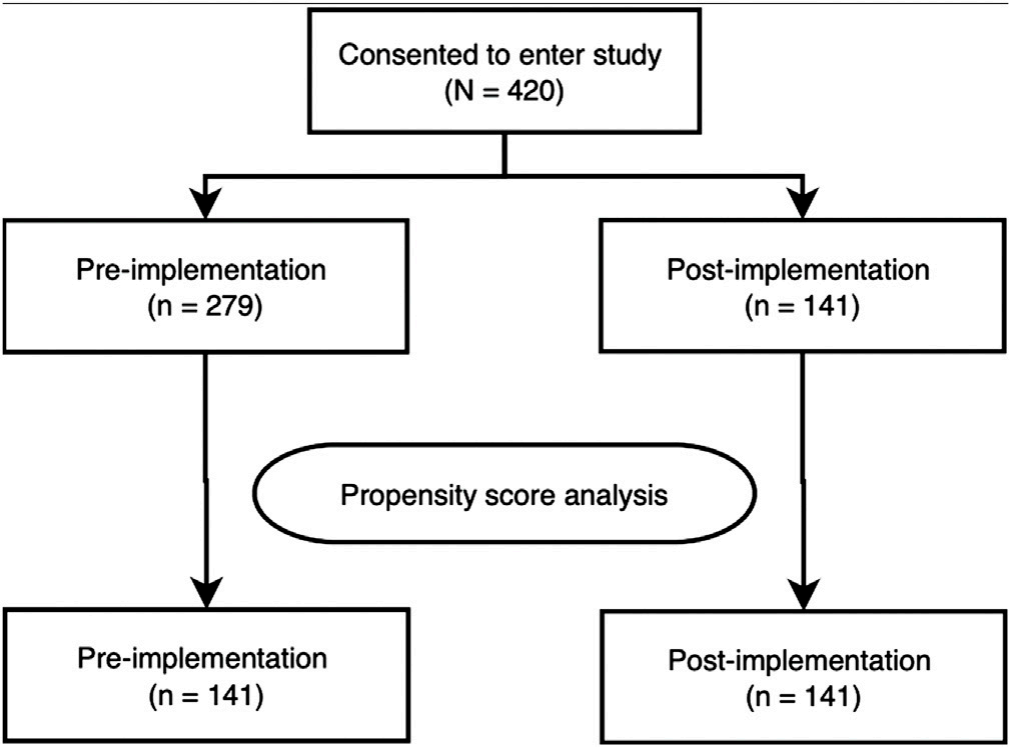
Participant flow diagram.

**FIGURE 2:**
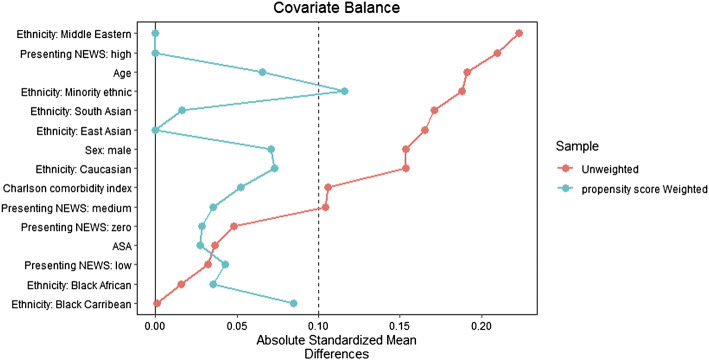
Love plot depicting covariate balance with standardized mean differences following propensity score matching.

The 1:1 matching method matched the post-implementation group (n = 141) with the same number of subjects from the pre-implementation group (n = 141) with the remaining unmatched samples dropped (n = 138, Figure 1). After matching, only ethnicity: Minority Ethnic and admission type (i.e., elective or emergency) remained imbalanced. However, given the low number of participants across these variables, and the overall improvement in other ethnicity categories, this difference was not deemed meaningful. Baseline demographics of the unmatched and matched cohorts are presented in Table 1. The standardised mean differences of all covariates are displayed as a love plot in Figure 2. The median age of the entire cohort was 52 (range: 18–95) years and the median duration of wearing the sensor was 1.3 (interquartile range: 0.7–2.0) days.

**TABLE 1:** Baseline demographics before and after propensity score matching.

Characteristic	Before Propensity Score Matching					After Propensity Score Matching		
	N	Pre-implementation, N = 279^*1*^	Post-implementation, N = 141^*1*^	*p*-value^*2*^	N	Pre-implementation, N = 141^*1*^	Post-implementation, N = 141^*1*^	*p*-value^*2*^
**Age**	420	51 (35–66)	55 (36–73)	0.061	282	51 (39–71)	55 (36–73)	0.54
**Sex**	420	—	—	0.14	282	—	—	0.55
F		154 (55)	67 (48)	—	—	72 (51)	67 (48)	—
M		125 (45)	74 (52)	—	—	69 (49)	74 (52)	—
**Ethnicity**	420	—	—	0.008	282	—	—	0.71
Black African	—	11 (3.9)	6 (4.3)	—	—	5 (3.5)	6 (4.3)	—
Black Carribean	—	2 (0.7)	1 (0.7)	—	—	0 (0)	1 (0.7)	—
Caucasian	—	193 (69)	87 (62)	—	—	92 (65)	87 (62)	—
East Asian	—	5 (1.8)	0 (0)	—	—	—	—	—
Middle Eastern	—	9 (3.2)	0 (0)	—	—	—	—	—
Minority ethnic	—	5 (1.8)	9 (6.4)	—	—	5 (3.5)	9 (6.4)	—
South Asian	—	54 (19)	38 (27)	—	—	39 (28)	38 (27)	—
BMI	276	27 (23–31)	28 (25–31)	0.63	177	27 (23–32)	28 (25–31)	0.83
**ASA**	420	—	—	0.51	282	—	—	0.24
1		61 (22)	30 (21)	—	—	28 (20)	30 (21)	—
2		161 (58)	83 (59)	—	—	84 (60)	83 (59)	—
3		47 (17)	19 (13)	—	—	26 (18)	19 (13)	—
4		10 (3.6)	9 (6.4)	—	—	3 (2.1)	9 (6.4)	—
**Charlson Comorbidity index**	420	1.00 (0.00–3.00)	1.00 (0.00–3.00)	0.27	282	1 (0–3)	1 (0–3)	0.65
**Presenting NEWS severity**	420	—	—	0.12	282	—	—	0.91
zero	—	120 (43)	64 (45)	—	—	62 (44)	64 (45)	—
low	—	145 (52)	71 (50)	—	—	74 (52)	71 (50)	—
medium	—	6 (2.2)	6 (4.3)	—	—	5 (3.5)	6 (4.3)	—
high	—	8 (2.9)	0 (0)	—	—	0 (0)	0 (0)	—
**Admissions**	420	—	—	0.001	282	—	—	0.006
elective	—	2 (0.7)	10 (7.1)	—	—	1 (0.7)	10 (7.1)	—
emergency	—	277 (99)	131 (93)	—	—	140 (99)	131 (93)	—

1 Median (IQR); n (%).

2 Wilcoxon rank sum test; Pearson’s Chi-squared test; Fisher’s exact test.

### Response to Alerts and Clinical Outcomes

Overall, 78 alerts were generated for 46 participants. Of which, 58 alerts (33 participants) were actioned. Nursing staff acknowledged generated alerts through a designated mobile application on provided devices, a detailed breakdown of responses by abnormal vital parameter is tabulated in Table 2 for actioned alerts. Members of the research team met weekly with ward managers and senior nurses to monitor the workflow change and feedback previous alerting acknowledgement times as well as gather barriers to change and will be described separately. Briefly, however, themes relating to inappropriate resources (i.e., poor staffing levels and sudden unincentivized workflow changes) were noted. Furthermore, regular training sessions for the nursing staff to encourage use of mobile devices in responding to digital alerts was provided.

**TABLE 2:** Action taken following abnormal vital sign (heart rate, respiratory rate, temperature) alert.

Action Taken	Heart Rate	Respiratory Rate	Temperature	N
Full set of observations repeated	2 (3.4%)	12 (21%)	5 (8.6%)	19 (33%)
Initiated Sepsis Pathway	1 (1.7%)	0 (0%)	4 (6.9%)	5 (8.6%)
No Action Taken	4 (6.9%)	8 (14%)	1 (1.7%)	13 (22%)
Participant clinically well after review	0 (0%)	10 (17%)	0 (0%)	10 (17%)
Reapplied Electrodes	1 (1.7%)	6 (10%)	1 (1.7%)	8 (14%)
Refer to Senior clinician	1 (1.7%)	0 (0%)	2 (3.4%)	3 (5.2%)
N, n (%)	9 (16%)	36 (62%)	13 (22%)	58 (100%)

**FIGURE 3:**
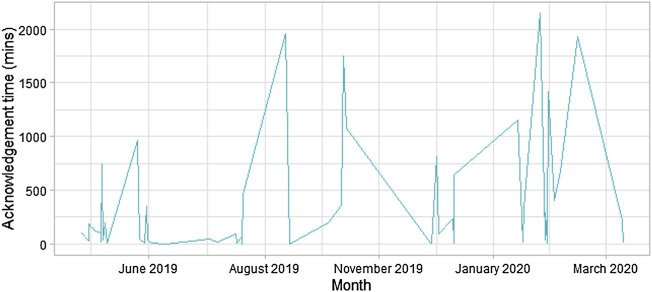
Time series displaying the alert acknowledgement time by healthcare staff.

The median alert acknowledgement time was 111 (range: 1–2,146) minutes. The sizeable variation in alert acknowledgement time has been plotted in Figure 3.

Clinical events have been summarised in Table 3. Overall, planned (odds ratio (OR): 0.49; 95% CI 0.02–5.20) and unplanned (OR: 0.49; 95% CI 0.02–5.20) intensive care admissions, 28-days mortality (OR 0.99; 95% CI 0.04–25.3), and length of stay (incidence rate ratio: 1.03; 95% CI 0.92–1.14) were similar across both cohorts, following propensity score matching.

**TABLE 3:** Summary of outcome measures before and after propensity score matching.

Outcome	Before Propensity			Score Matching			After Propensity		Score Matching	
	OR (95%CI)		*p*-Value	IRR (95% CI)	*p*-Value		OR (95%CI)	*p*-Value	IRR (95% CI)	*p*-Value
ITU admissions (planned)	0.33 (0.02–1.93)	0.30		—		—	0.49 (0.02–5.20)	0.57	—	—
ITU admissions (Unplanned)	0.39 (0.02–2.47)	0.40		—		—	0.49 (0.02–5.20)	0.57	—	—
28D mortality	0.99 (0.05–10.5)	0.99		—		—	0.99 (0.04–25.3)	0.99	—	—
Length of stay	—	—		1.04 (0.95–1.13)		0.44	—	—	1.03 (0.92–1.14)	0.63

OR: oa2dds ratio; CI: confidence interval; IRR: incidence rate ratio.

## Discussion

This study demonstrated similar outcome measures (i.e., hospital LOS, mortality, and ITU admissions) with the use of wearable sensors and alerting systems across an acute surgical cohort in our institution. In contrast, a recent meta-analysis reported that digital alerting reduced hospital LOS (Iqbal et al., 2021c). However, the majority of constituent studies were of low quality; moreover, the considerable variation in time taken to respond to an alert by healthcare staff, rapid turnover of participants resulting in the short duration of sensor use, and the premature conclusion of our trial may additionally explain our findings. Perceptions of healthcare staff involved in this study were largely favourable of digital solutions given the potential improvements for patient safety and reduced staff burden, despite no previous experience with telemetry or digital solutions (Joshi et al., 2022). However, this contradicts our findings of prolonged response times. This is likely to be multifactorial including changing culture of adopting innovation; inadequate resources (both time and available training) for healthcare professionals; winter-bed pressures; but, particularly in this study, the perceived usefulness by healthcare professionals and the impact of sensing technology in its current state was deemed as unincentivized additional labour given rota shortages and absence of permanent staff (Joshi et al., 2022).

Alerts generated for abnormal respiratory rates were common in our cohort, yet none of the participants required escalation for senior review and were deemed well. This false alarm phenomenon has been reported elsewhere and is a recognised limitation of impedance pneumography as a technique utilised by the sensor to measure respiratory rate (Wong et al., 2009; Downey et al., 2019). Furthermore, this may affect the perceived usefulness of sensing systems, contributing to disengagement from healthcare staff and prolonged response times, as noted in our study. Machine learning approaches have the potential to improve artefact detection (Moeyersons et al., 2021); further work is required to determine if the implementation of classification models, convolutional neural networks, or heuristic algorithms within wearable sensors can improve the accuracy of respiratory rate readings. Conversely, alerts for abnormal heart rate and temperature resulted in senior review and initiation of further treatment but the proportion of meaningful actions taken were low, suggesting that further optimisation for parameter thresholds was required and warrants further exploration. Despite this, wearable sensors were well perceived by the participants, with enhanced feelings of patient safety, comfort, and centralised monitoring (Joshi et al., 2021), in keeping with the literature (Downey et al., 2018a; Weenk et al., 2020).

Implementation of digital technologies has historically been challenging, largely due to human and organisational factors. Parallels can be drawn from the introduction and implementation of electronic health records (EHR). Although global evidence has demonstrated the improved record quality, increased administrative efficiency, and enhanced quality of care (such as reduced medication errors and higher guideline adherence) with EHR use (Nguyen et al., 2014; Campanella et al., 2016; Baumann et al., 2018), implementation across complex hospital systems has been challenging, particularly in the United Kingdom (Wachter, 2016).

One facet of complexity science, a physician’s perception of uncertainty, was linked to poor EHR use across a diverse range of medical specialties (Lanham et al., 2014). Changes to workflow, time constraints, and lack of user involvement have all been reported as major barriers to successful implementation (Nguyen et al., 2014). To draw parallels, although sensors are increasingly able to measure multiple vital parameters, not all parameters can be recorded by all sensor systems and some parameters remain intermittent (i.e., blood pressure). As a result, there remains a reliance on multiple methods for adequate early warning score calculations. This can be a source of frustration for healthcare staff and for patients requiring multiple modalities for monitoring, reducing overall healthcare efficiency.

One study reported the experience of alert fatigue for healthcare staff; the burdensome nature to carry additional devices; and inadequate training for nursing staff to interpret continuous data as barriers to successful adoption of digital alerting (Weenk et al., 2020). These factors are likely to have contributed towards the substantial variation in alert acknowledgement by healthcare staff noted in this trial. Introduction of health policies and legislation, in conjunction with apt resources, may be a meaningful way to facilitate workflow change. The passage of the Health Information Technology for Economic and Clinical Health Act in 2009 and the Meaningful Use policy, helped overcome the previously stagnant adoption of EHR (Cohen, 2016; Health IT, 2016; FastStats, 2019).

Although our pragmatic design highlights real-world applicability, it presents inherent limitations. Firstly, this study used PSM to adjust for several important variables, however additional relevant information that could affect outcomes may be missing. Secondly, the lack of randomisation makes establishing causal relationships difficult. Furthermore, clinical research was difficult to mandate resulting in an unincentivized workflow change to healthcare staff, a limitation in effectively testing the efficacy of digital alerting systems in the real world. The fast turnover of the acute surgical unit, in hindsight, likely contributed to the short length of stay and duration of sensor limiting the inference of our outcome measures and risking attrition bias. The pandemic has accelerated the adoption of digital technologies and altered perceptions favourably towards their adoption (Golinelli et al., 2020; Whitelaw et al., 2020); as this trial concluded prematurely, at the onset of COVID-19, our work may be perceived differently if it were repeated in the current climate. Additionally, the early conclusion of this work has likely resulted in the underpowering of our trial. Lastly, digital literacy, human and system factors (e.g., staffing) play a significant contributory role in successful adoption of novel digital solutions and were not fully examined in this trial.

Future research should seek to include information system evaluation frameworks that test implementation attributes, strategies, and organisational aspects during piloting of digital solutions, particularly as the pandemic has altered attitudes towards digitisation (Golinelli et al., 2020; Whitelaw et al., 2020). Indeed, sensors and alerting systems have been met favourably when used for remote monitoring of individuals suspected of COVID-19 (Iqbal et al., 2021a). Furthermore, further refinements to wearable sensors are required to offer monitoring of all vital parameters, this may aid workflow improvement and adoption. Following this, trials should test a variety of alerting mechanisms that are actionable by multiple healthcare professionals of different seniority. Our work has highlighted the need to consider organisational, system, and human factors when implementing novel digital solutions. For optimal integration, we recommend the involvement of healthcare staff during trial design when changes to workflows are expected with consideration of mandated changes; PDSA cycles for model of improvement may be a useful tool in such settings, allowing for regular evaluations (Leis and Shojania, 2017). Testing novel digital solutions through the quality improvement measures may be a more effective means of testing their real-world efficacy. Furthermore, we recommend ensuring adequate training (and re-training) is provided with the introduction of new technologies and that a variety of healthcare professionals are included in an area of the hospital with longer inpatient stay. This trial may have been perceived differently if junior doctors were responsible for acknowledging clinical alerts.

In conclusion, wearable sensors and alerting systems remain an area of growing interest, particularly in the pandemic era; as the growth of development increases, an alternative workflow favouring digitisation is likely to take precedent. For successful implementation and optimisation of novel systems, human and organisational factors should be tested in conjunction of digital solution deployment where further work should be conducted.

## Data Availability

The datasets generated during and/or analysed during the current study are available from the corresponding author on reasonable request.
